# Circulating Metabolomic and Lipidomic Signatures Identify a Type 2 Diabetes Risk Profile in Low-Birth-Weight Men with Non-Alcoholic Fatty Liver Disease

**DOI:** 10.3390/nu15071590

**Published:** 2023-03-24

**Authors:** Line O. Elingaard-Larsen, Sofie O. Villumsen, Louise Justesen, Anne Cathrine B. Thuesen, Min Kim, Mina Ali, Else R. Danielsen, Cristina Legido-Quigley, Gerrit van Hall, Torben Hansen, Tarunveer S. Ahluwalia, Allan A. Vaag, Charlotte Brøns

**Affiliations:** 1 Steno Diabetes Center Copenhagen, 2730 Herlev, Denmark; sofie.olund.villumsen.02@regionh.dk (S.O.V.); charlotte.broens.01@regionh.dk (C.B.); 2 Novo Nordisk Foundation Center for Basic Metabolic Research, Faculty of Health and Medical Sciences, University of Copenhagen, 2100 Copenhagen, Denmark; 3 Department of Radiology, Rigshospitalet, 2100 Copenhagen, Denmark; 4 Clinical Metabolomics Core Facility, Clinical Biochemistry, Rigshospitalet, 2100 Copenhagen, Denmark; 5 Department of Biomedical Sciences, Faculty of Health and Medical Science, University of Copenhagen, 2100 Copenhagen, Denmark; 6 The Bioinformatics Center, Department of Biology, University of Copenhagen, 2100 Copenhagen, Denmark; 7 Lund University Diabetes Centre, Department of Clinical Sciences, Lund University, 214 28 Malmö, Sweden

**Keywords:** low-birth-weight, liver fat, non-alcoholic fatty liver disease, metabolomics, lipidomics, type 2 diabetes

## Abstract

The extent to which increased liver fat content influences differences in circulating metabolites and/or lipids between low-birth-weight (LBW) individuals, at increased risk of type 2 diabetes (T2D), and normal-birth-weight (NBW) controls is unknown. The objective of the study was to perform untargeted serum metabolomics and lipidomics analyses in 26 healthy, non-obese early-middle-aged LBW men, including five men with screen-detected and previously unrecognized non-alcoholic fatty liver disease (NAFLD), compared with 22 age- and BMI-matched NBW men (controls). While four metabolites (out of 65) and fifteen lipids (out of 279) differentiated the 26 LBW men from the 22 NBW controls (*p* ≤ 0.05), subgroup analyses of the LBW men with and without NAFLD revealed more pronounced differences, with 11 metabolites and 56 lipids differentiating (*p* ≤ 0.05) the groups. The differences in the LBW men with NAFLD included increased levels of ornithine and tyrosine (P_FDR_ ≤ 0.1), as well as of triglycerides and phosphatidylcholines with shorter carbon-chain lengths and fewer double bonds. Pathway and network analyses demonstrated downregulation of transfer RNA (tRNA) charging, altered urea cycling, insulin resistance, and an increased risk of T2D in the LBW men with NAFLD. Our findings highlight the importance of increased liver fat in the pathogenesis of T2D in LBW individuals.

## 1. Introduction

Low-birth-weight (LBW) is a marker of an adverse fetal environment and is associated with an increased risk of developing type 2 diabetes (T2D) [1,2]. We recently reported that 20% of non-obese (BMI < 30 kg/m^2^) LBW individuals with a mean age of 38 years exhibit previously unrecognized non-alcoholic fatty liver disease (NAFLD), supporting the hypothesis of ectopic fat deposition as a key pathophysiological feature underlying development of T2D in individuals born with a LBW [3,4,5,6]. The LBW individuals with NAFLD were characterized by elevated hepatic insulin resistance, fasting levels of insulin, *C*-peptide, and triglycerides (TG), as well as elevated plasma levels of leptin and GLP-1, compared with both LBW and normal-birth-weight (NBW) individuals without NAFLD [3]. As an exception, plasma adiponectin levels were decreased to the same extent in LBW individuals with and without NAFLD compared with NBW individuals [3]. Nevertheless, the results collectively suggested that increased liver fat and NAFLD may be on the critical path to T2D development in LBW subjects.

Analysis of circulating metabolites and lipids can provide important insights into the metabolic signatures of NAFLD [7]. Different metabolite patterns have been reported in studies performed on individuals with NAFLD; however, a common denominator in NAFLD are increased levels of fasting concentrations of circulating amino acids [7,8,9,10]. Lipidomics studies have demonstrated that NAFLD is associated with an increase in plasma levels of triglycerides with low numbers of double bonds and short carbon-chain length, mimicking structural characteristics previously reported to be associated with insulin resistance and risk of T2D [11,12,13]. Furthermore, lysophosphatidylcholines (LPC) and polyunsaturated fatty acids containing phospholipids have been shown to be negatively associated with liver fat, and together with saturated TGs, this lipid signature has been shown to be predictive of NAFLD [12].

In a meta-analysis of seven cohorts, lower birth weight was associated with increased levels of aromatic and branched-chain amino acids as well as of saturated and monounsaturated fatty acids [14]. Furthermore, a lower birth weight was associated with markers of low-grade inflammation and liver dysfunction indicating a higher risk of cardiometabolic disease [14]. The finding of altered liver function markers supports the emerging recognition of an increased risk of ectopic liver fat deposition including overt NAFLD in LBW subjects.

However, whether non-obese LBW subjects with screen-detected NAFLD exhibit similar dysmetabolic traits as patients diagnosed with NAFLD in the clinics due to symptoms or elevated liver function tests is unknown. Moreover, the extent to which elevated hepatic fat content and/or overt NAFLD may contribute to or explain changes of the metabolome and/or lipidome in LBW subjects with increased risk of developing T2D remains to be explored. Thus, the main objective of the current study was to identify differential metabolomic and/or lipidomic signatures in healthy, early-middle-aged non-obese LBW subjects with and without NAFLD, compared with matched NBW controls without NAFLD.

## 2. Materials and Methods

### 2.1. Study Participants

The study included 48 healthy Caucasian males born at gestational weeks 39–41 between 1979 and 1980 and recruited from the Birth Registry at the Danish Health Data Authority, as described previously [3]. In total, 26 individuals were born with a birth weight < 10th percentile (LBW) and 22 individuals were age- and BMI-matched controls born with a birth weight between the 50th and 90th percentile (NBW). Exclusion criteria included diabetes in first-degree relatives, disease and medication known to affect outcomes, BMI > 30 kg/m^2^, self-reported physical activity > 10 h/week, and alcohol intake above the national recommendation. The study was approved by the ethical committee of the Capital Region of Denmark (H-4-2014-128) and carried out according to the Declaration of Helsinki II, and written informed consent was obtained from all participants. ClinicalTrials.gov identifier: NCT02982408.

### 2.2. Study Design

The participants were standardized with regards to diet, alcohol intake, and physical activity for three days prior to examination. Fasting blood samples were obtained after a 10 h overnight fast and stored at −80 °C for later use. Clinical examinations, including magnetic resonance spectroscopy (MRS) of hepatic liver fat content and isotope tracers for glucose kinetics, were performed as described previously [3]. A priori, healthy participants were included whose NAFLD status was unknown. However, the critical finding of MRS screen-detected NAFLD in LBW subjects led us to perform a post hoc division of the population into three groups. Further downstream analyses included four comparisons: the initial LBW vs. NBW comparison together with three subgroup comparisons.

### 2.3. Untargeted Serum Metabolomics

Fasting serum samples were analyzed using two-dimensional gas chromatography coupled to time-of-flight mass spectrometry (GC×GC-TOF-MS) (Leco Corp., St. Joseph, MI, USA) [15,16] (Supplementary Methods). In all, 65 circulating metabolites, including fatty acids, carbohydrates, amino acids, and other sub-classes (Table S1), could be semiquantified and were known species out of 466 measured. The 65 semiquantified metabolites were included in the current study.

### 2.4. Untargeted Serum Lipidomics

Fasting serum samples were analyzed in a random order in positive and negative electrospray ionization modes using ultrahigh-performance liquid chromatography quadrupole time-of-flight mass spectrometry (UHPLC-Q-TOF-MS) (Agilent Technologies, Santa Clara, CA, USA) [17,18,19,20,21,22] (Supplementary Methods). A total of 279 lipids were semiquantified into the following lipid classes: triglycerides (TG), phosphatidylcholines (PC), phosphatidylethanolamines (PE), phosphatidic acid (PA), phosphatidylglycerol (PG), lysophosphatidylcholines (LPC), sphingolipids (SM), cholesteryl esters (CE), and ceramides (CER) (Table S2).

### 2.5. Statistical Analyses

Quality control of metabolomics and lipidomics data was completed prior to the analyses using R 4.1.0 (R Foundation for Statistical Computing) (Supplemental Methods).

Differential abundances in the metabolomics and lipidomics data were analyzed in four comparisons between the three groups, as follows: LBW (all) vs. NBW; LBW with NAFLD (LBW w/NAFLD) vs. NBW; LBW without NAFLD (LBW w/o NAFLD) vs. NBW; and LBW w/NAFLD vs. LBW w/o NAFLD. The four comparisons were analyzed using linear regression models on log2-transformed and scaled omics data (R-package limma), adjusting for multiple testing with false discovery rate (FDR) (P_FDR_) using the Benjamini–Hochberg method. The log2 transformation demonstrates the ratio between the mean metabolite abundance for two groups and is referred to as the log fold change. Metabolites and lipids with P_FDR_ ≤ 0.1 were considered significant, whereas metabolites and lipids with *p* ≤ 0.05 (unadjusted) were considered suggestive. The models were not further adjusted, as the NBW and LBW groups were matched on sex, age, and BMI. Principal component analysis (PCA) plots based on metabolites and lipids with differential abundance between groups (P_FDR_ ≤ 0.1 and *p* ≤ 0.05) were generated (R-package broom) to examine how individuals differed from each other within each group. Furthermore, a heatmap with the log2-normalized abundances of the 11 metabolites of interest (P_FDR_ ≤ 0.1 and *p* ≤ 0.05), ordered by the three groups, was produced to illustrate the differential abundances (R-package ComplexHeatmap). A Pearson correlation analysis between the first principal component (PC1) and the second (PC2) and a panel of selected clinical variables associated with specific metabolic traits was conducted (R-package PCAtool) and FDR corrected. Volcano plots were created based on the lipidomics data to illustrate the differentially abundant lipids between the groups. A structural composition analysis of the lipids, assessing the importance of the lipid fatty acid carbon-chain lengths and the number of double bonds was performed (R-package LipidomeR) comparing both LBW w/o NAFLD and LBW w/NAFLD with NBW subjects.

### 2.6. Pathway Enrichment Analysis

All circulating metabolites, including their *p*-values and FDR values obtained from the differential abundance analyses (*p* ≤ 0.05), together with the Human Metabolome Data Base (HMDB) and Kyoto Encyclopedia of Genes and Genomes (KEGG) annotation IDs, were used to map enriched pathways using the Ingenuity Pathway Analysis application (IPA, by Qiagen). IPA generated enriched metabolic pathways based on differences between the groups.

### 2.7. Correlation Network Analysis

Correlations (Spearman’s, [rho < −0.4; rho > 0.4], P_FDR_ ≤ 0.1) between identified key metabolites (*n* = 11) and all lipids (*n* = 279) in the NBW individuals were calculated (R-package correlation) [23]. The correlations were visualized by a network to identify coordinated metabolite–lipid patterns of the NBW control group, which was used as a null model (i.e., reference network). The nodes represented metabolites and lipids, and edges represented the correlations ([rho < −0.4; rho > 0.4], P_FDR_ ≤ 0.1) in the network created using Cytoscape 3.7.1 [24]. Analyses of network characteristics (i.e., topology and other properties) were carried out to ensure that the network properties differed from a random network’s properties. Lastly, we identified communities of metabolites and lipids sharing common structural characteristics (i.e., fatty-acid chain-length and number of double bonds) using the Leiden algorithm (R-package LeidenAlg) [25].

## 3. Results

### 3.1. Clinical Characteristics

The LBW individuals w/NAFLD had a significantly increased hepatic fat content with a median hepatic fat content of 9.45% compared with a hepatic fat content of 0.80% and 0.78% in LBW subjects w/o NAFLD and NBW controls, respectively (Table 1), as previously published [3]. Furthermore, 20% of the otherwise previously healthy LBW individuals had MRS screen-detected NAFLD [3]. The LBW subjects w/NAFLD showed differential metabolic changes compared with both LBW w/o NAFLD and NBW control men, including increased hepatic insulin resistance (*p* = 0.02), fasting plasma TGs (*p* = 0.03), cholesterol (*p* = 0.05), and *C*-peptide (*p* = 0.02) [3].

### 3.2. Serum Metabolomics

Out of the 65 semiquantified serum metabolites, four metabolites were differentially abundant (*p* ≤ 0.05) between the LBW (all) and the NBW groups (Table 2). These included higher levels of leucine (*p* = 0.02), alpha-tocopherol (*p* = 0.03), and cholesterol (*p* = 0.04), and lower levels of hippuric acid (*p* = 0.04) in the LBW subjects.

Seven metabolites differed significantly between the LBW sub-group w/NAFLD and the NBW group before multiple-testing corrections (Table 2), including increased levels of ornithine (*p* = 0.001), tyrosine (*p* = 0.007), citrulline (*p* = 0.02), leucine (*p* = 0.02), and 2-oxoisovaleric acid (*p* = 0.03) in the LBW w/NAFLD subjects. Additionally, linoleic acid (*p* = 0.02) and arachidonic acid (*p* = 0.05) levels were significantly decreased in the LBW w/NAFLD subjects compared with the NBW subjects.

Two metabolites differentiated the LBW w/o NAFLD from the NBW subjects before FDR adjustment, namely a decrease in hippuric acid (*p* = 0.01) and an increase in alpha-tocopherol levels (*p* = 0.04) in the LBW w/o NAFLD subjects (Table 2).

When comparing the LBW individuals w/NAFLD with the LBW individuals w/o NAFLD, six metabolites differentiated the groups, including significantly increased levels of tyrosine (*p* = 0.001), ornithine (*p* = 0.003), citrulline (*p* = 0.02), and alpha-ketoglutaric acid (*p* = 0.04) in the LBW subjects w/NAFLD. Moreover, linoleic (*p* = 0.03) and arachidonic (*p* = 0.03) acid levels were decreased among the LBW subjects w/NAFLD (Table 2).

However, after multiple-testing corrections, only ornithine (P_FDR_ = 0.08) remained significant for the LBW w/NAFLD vs. the NBW subjects, while tyrosine (P_FDR_ = 0.07) and ornithine (P_FDR_ = 0.1) remained significant for the LBW w/NAFLD vs. LBW w/o NAFLD groups (Table 2).

**Figure 1:**
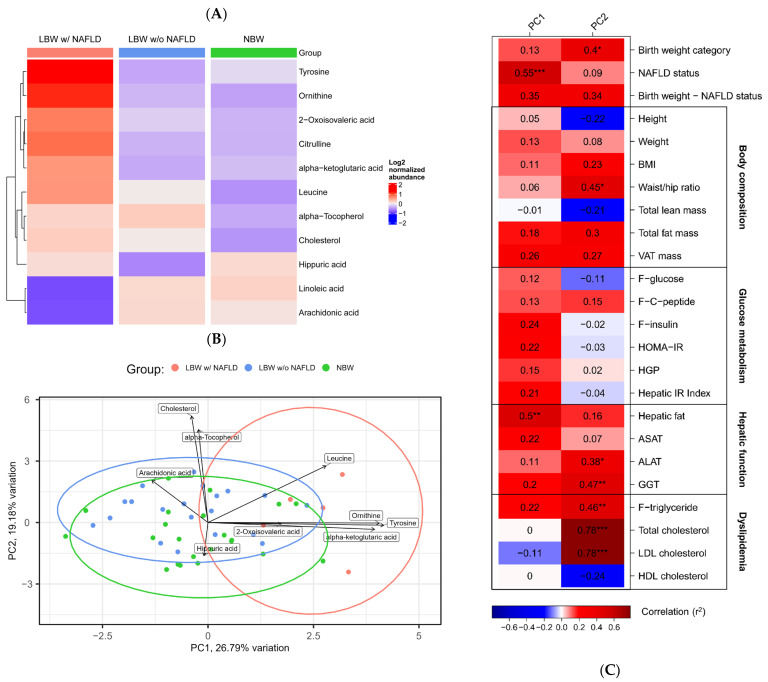
Metabolomics analysis of NBW controls and LBW with and without NAFLD identify key metabolites differentiating the 3 groups (*n* = 22, *n* = 21 and *n* = 5, respectively). (**A**) Heatmap of the log2 normalized abundances of the 11 distinct serum metabolites in the LBW w/NAFLD, LBW w/o NAFLD and NBW groups. Red color indicates increased abundance, blue color indicates decreased abundance. (**B**) PCA plot based on the 11 distinct metabolites. Each dot represents an individual. PC1 and PC2 explained ~46% of the total variance (PC1 = 26.79% and PC2 = 19.18%). The ellipses illustrate the 95% confidence interval. Metabolites with loadings above 0.1 (negative or positive) are labeled. (**C**) An Eigencor plot illustrating correlations between panels of clinical variables associated with a specific metabolic trait and PC1 and PC2 from the PCA (Pearson, r^2^ > 0.38), * (P_FDR_ ≤ 0.1), ** (P_FDR_ ≤ 0.01), and *** (P_FDR_ ≤ 0.001). Red color indicates positive correlation, blue color indicates negative correlation.

In total, 11 differential metabolites were identified (*p* ≤ 0.05), before multiple-testing corrections, from the four differential abundance analyses as shown in Table 2, also referred to as suggestive findings. Of these metabolites, ornithine and tyrosine remained significant even after multiple-testing corrections for the LBW subjects w/NAFLD. The log2 and Z-score metabolite abundances of the differential serum metabolites are shown in Figures S1–S4. Overall, a trend of elevated circulating amino acid levels and lower fatty acid levels was seen in the LBW subjects w/NAFLD compared with both the NBW and the LBW w/o NAFLD subjects across analyses, as illustrated in the heatmap (Figure 1A).

Group separations based on the 11 differential serum metabolites are shown in the PCA plot (Figure 1B). Component loadings retained from PC1 included tyrosine, ornithine, and alpha-ketoglutaric acid, mainly separating the LBW subjects w/NAFLD from the other two groups. Loadings retained from PC2, including cholesterol, alpha-tocopherol, and hippuric acid, mainly separated the LBW w/o NAFLD from the NBW subjects.

Correlations between clinical variables associated with body composition, glucose metabolism, hepatic function, dyslipidemia, and PC1 and PC2 (Pearson, P_FDR_ ≤ 0.1, r^2^ > 0.38) are illustrated in Figure 1C. PC1 correlated with NAFLD status (P_FDR_ ≤ 0.001, r^2^ = 0.55) and hepatic fat content (P_FDR_ ≤ 0.01, r^2^ = 0.5), whereas PC2 correlated with birth weight category (P_FDR_ ≤ 0.1, r^2^ = 0.4), waist/hip ratio (WHR) (P_FDR_ ≤ 0.1, r^2^ = 0.45), and levels of alanine aminotransferase (ALAT) (P_FDR_ ≤ 0.1, r^2^ = 0.38), gamma-glutamyltransferase (GGT) (P_FDR_ ≤ 0.01, r^2^ = 0.47), triglyceride (P_FDR_ ≤ 0.01, r^2^ = 0.46), total cholesterol (P_FDR_ ≤ 0.001, r^2^ = 0.78), and LDL cholesterol (P_FDR_ ≤ 0.001, r^2^ = 0.78) (Figure 1C).

### 3.3. Pathway Enrichment Analyses

Based on the 65 identified metabolites, IPA predicted the transfer RNA (tRNA) charging pathway as the top canonical pathway downregulated among the LBW w/NAFLD subjects compared with the other groups. Here, at least 75% of the examined amino acids (including leucine and tyrosine) were upregulated in the LBW w/NAFLD subjects compared with both the NBW and the LBW w/o NAFLD groups (P_FDR_ ≤ 0.1; Table S3). Additional enriched pathways in the LBW subjects w/NAFLD included the proline biosynthesis pathway, the sirtuin signaling pathway, the super pathway of citrulline metabolism, and the tyrosine degradation pathway. Furthermore, the upstream regulator mTOR (mammalian target of rapamycin) was suggested to be inhibited in the LBW w/NAFLD subjects compared with the NBW controls, whereas mitochondrial UCP2 (uncoupling protein 2) appeared to be inhibited among the LBW subjects both w/NAFLD and w/o NAFLD, compared with the NBW controls (Table S4).

### 3.4. Serum Lipidomics

**Figure 2:**
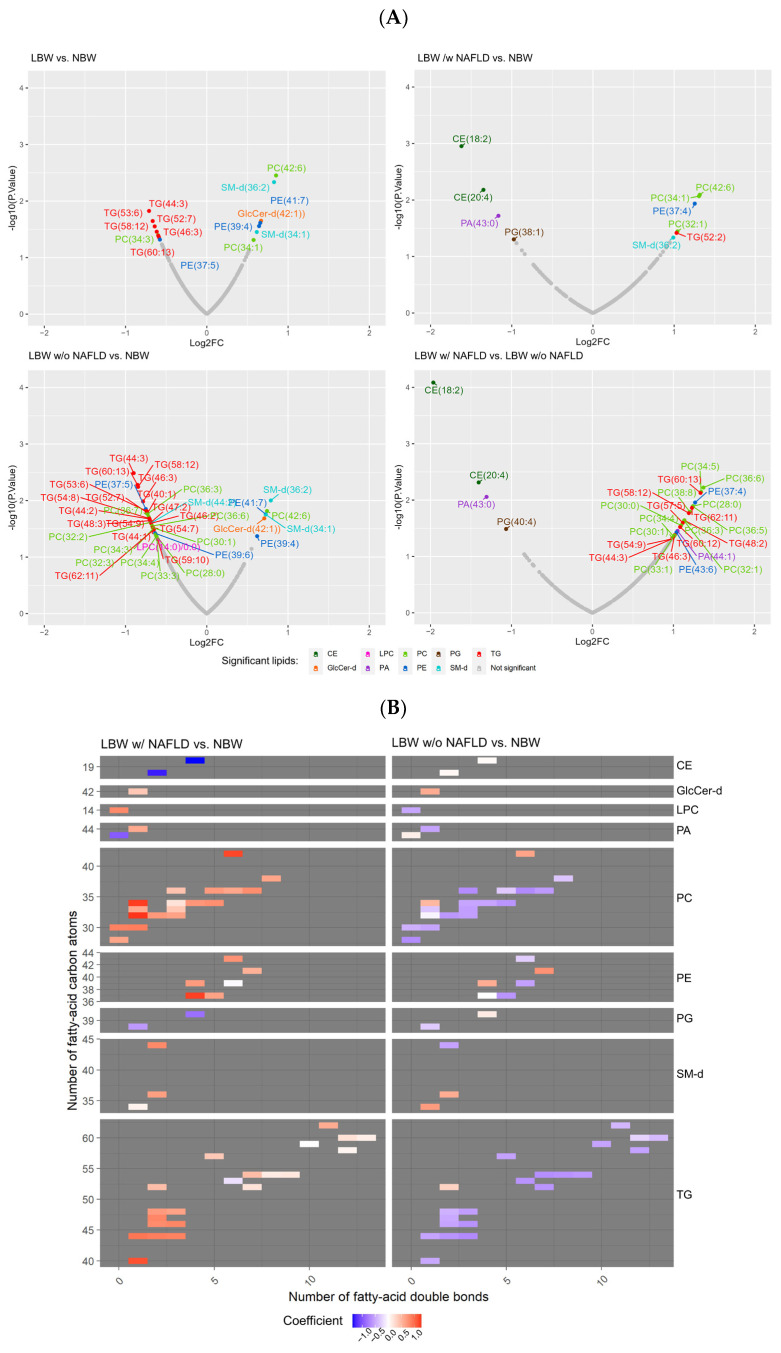
Lipidomics analyses revealed differences in lipid subclasses and lipid composition in LBW subjects with NAFLD compared with NBW subjects and LBW subjects w/o NAFLD. (**A**) Volcano plots illustrating the differentially abundant lipid species across the four abundance analyses. Log fold changes (Log2FC) are plotted on the *x*-axis and log10(*p*-value) on the *y*-axis. All plots use a *p*-value cutoff of *p* ≤ 0.05. Only significant lipids are denoted with their corresponding name. Lipid sub-classes are color-coded as shown in the legend. (**B**) Structural heatmaps for each lipid class including the 56 lipids of interest. Each lipid species is shown as a rectangle and the color shows the abundance difference between LBW subjects w/NAFLD compared with NBW subjects (left column) and LBW subjects w/o NAFLD compared with NBW subjects (right column). Red indicates an increased abundance and blue a decreased abundance. Lipids are organized by the level of saturation (number of double bonds) on the *x*-axis and the chain length (total number of carbon atoms) on the *y*-axis. To estimate the length of each fatty acid chain, the summarized number is divided by the number of fatty acid chains of the lipid species (e.g., TGs are divided by three, as this group contains three fatty acid chains).

All 279 lipid species were used in the differential abundance analyses between the groups. In total, 15 serum lipid species were differentially abundant (*p* ≤ 0.05) between the LBW (all) and the NBW subjects before correcting for multiple testing (Table S5). Of these, seven lipid species were slightly increased ([0.5, 0.8] logFC), including GlcCer, PC, PE, and SM, whereas eight lipids showed slightly lower levels ([−0.5, −0.7] logFC), primarily TGs (6 lipids), but also one PC and one PE (Figure 2A and Figure S5).

Comparing the LBW w/NAFLD subjects with the NBW controls, 10 differentially abundant lipids were identified. Six species from the PC, PE, SM, and TG subclasses were multifold increased (up to 13 in logFC, *p* ≤ 0.05; Table S5), while four lipid species (the CE, PA, and PG subclasses) were decreased in the LBW w/NAFLD group. CEs showed the most significantly multifold decreased levels (down to −16 in logFC, *p* = 0.001) (Figure 2A and Figure S6, Table S5).

A total of 37 lipids differed in abundance (*p* ≤ 0.05) in the LBW w/o NAFLD subjects compared with the NBW group, before FDR adjustment (Table S5). Most of these differential lipids (*n* = 31) displayed a slight decrease in abundance in the LBW w/o NAFLD group ([−0.6,−0.8] logFC), with the vast majority belonging to the PC and TG subclasses, as well as a few PEs, one SM, and one LPC. Only six lipid species displayed slight increased levels ([0.6, 0.7] logFC) in the LBW w/o NAFLD subjects, including one CER, one PC, PEs, and SMs (Figure 2A and Figure S7).

Lastly, comparing the LBW group w/NAFLD with the LBW group w/o NAFLD, 27 lipids were differentially abundant (*p* ≤ 0.05) (Table S5, Figure S8). Twenty-three lipid species, of which the majority were classified as PCs and TGs, along with a few PEs and one PA, were highly abundant (up to 13 in logFC) in the LBW w/NAFLD group. CEs were the most decreased in abundance (down to −19 in logFC) of the four lipid species found to be decreased within the LBW w/NAFLD group, as illustrated in the Volcano plots (Figure 2A). Only CE(18:2) levels remained significant after multiple-testing corrections (P_FDR_ = 0.02).

Out of the 279 lipids, the four differential abundance analyses (described in Section 2.5) identified a total of 56 differential lipid species (*p* ≤ 0.05 threshold) (Table S5). Group separation based on these 56 lipids using PCA showed no clear separation of the groups (Figure S9).

Interestingly, when comparing the concentration of the different lipid species based on lipid structure (carbon-chain length and number of double bonds), the LBW w/NAFLD group showed a distinct pattern compared with the NBW subjects and the LBW subjects w/o NAFLD (Figure 2B). In the LBW subjects w/NAFLD, the level of PCs with double bonds between 0 and 7 and a carbon-chain length between 14 and 21 (total number of carbon atoms of 28–42) was increased compared with the NBW controls. Similarly, the number of TGs with double bonds between 1 and 3 and a carbon-chain length between 14 and 16 (total number of carbon atoms of 40–52) was higher in the LBW subjects w/NAFLD (Figure 2B, left panel). Conversely, the concentration of CEs with few double bonds was strongly decreased compared with the NBW controls. In contrast, LBW subjects w/o NAFLD showed decreased concentrations of PCs and TGs with few double bonds (0–7 and 1–3, respectively) and shorter carbon-chain lengths (14–21 and 14–16, respectively) (Figure 2B, right panel).

### 3.5. Correlation Network Analysis

**Figure 3:**
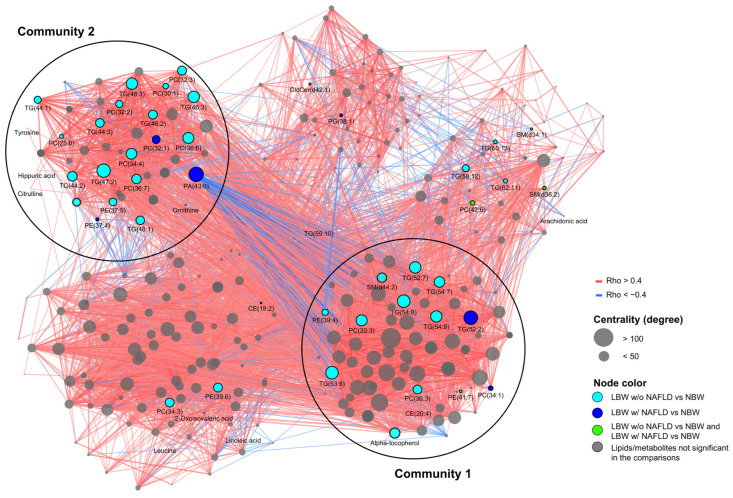
Correlation network of lipids and key metabolites. In the correlation network, nodes represent metabolites and lipids, and edges represent their correlations ([rho < −0.4; rho > 0.4], P_FDR_ ≤ 0.1). Red edges indicate positive correlations, and blue, negative correlations. The size of each node corresponds to its centrality; thus, the bigger the node, the more correlated with other nodes it is. Colored nodes represent metabolites/lipids found to differentiate in NBW vs. LBW w/o NAFLD (light blue), NBW vs. LBW w/NAFLD (dark blue), and in both NBW vs. LBW w/o NAFLD and LBW w/NAFLD vs. NBW (light green). The two most important communities (C1 and C2) are visualized with black circles and text.

The correlation network analysis identified patterns highlighting the relationship between key metabolomic and lipidomic profiles that were enriched for the LBW individuals w/NAFLD and w/o NAFLD compared with the NBW controls. We mapped differentially abundant metabolites (*n* = 11) and lipids (*n* = 56) onto the NBW reference network to identify significantly differing molecular patterns and communities between the LBW w/ and w/o NAFLD groups (Table 2 and Table S5, Figure 3). The NBW metabolite–lipid network included 290 nodes (representing the 11 metabolites and 279 lipids) and 5450 edges (each edge corresponding to a correlation between metabolites and lipids) (P_FDR_ < 0.1, [rho < −4; rho > 0.4]). Metabolites/lipids were clustered into communities, identifying functional groups of lipids having similar properties, and metabolites which were tightly connected due to shared regulatory processes [26]. Here, Community 1 (C1) was identified as the most central community (i.e., correlated) in the network, containing 17 out of all key metabolites/lipids, including cholesterol and alpha tocopherol, together with TGs, PCs, PEs, CEs, and SMs, whereas C2 contained 28 out of all key metabolites/lipids, including citrulline, tyrosine, ornithine, and hippuric acid, together with TGs, PCs, PAs, PEs, and LPCs. Most amino acids of interest clustered into C2 together with TGs of shorter and more saturated fatty acid chains, compared with the TGs in C1, which included longer and more polyunsaturated fatty acid chains. 

## 4. Discussion

We here demonstrate differences in metabolomics and lipidomics between LBW and NBW subjects, which appeared primarily to be attributed to the presence or absence of NAFLD in the LBW group. The LBW subjects w/NAFLD exhibited elevated levels of circulating amino acids, as well as increased abundance of PCs and TGs consisting of fatty acids with shorter chain length and fewer double bonds when compared with the NBW and LBW subjects without NAFLD. These differentially abundant circulating metabolites are suggested to be involved in urea and/or nitric oxide (NO) cycling, development of insulin resistance, and inflammation, and have furthermore previously been shown to be associated with NAFLD, fibrosis, and T2D [10,27]. To this end, the identified pattern of increased levels of shorter carbon chains and more saturated fatty acids in the LBW w/NAFLD subjects was previously associated with insulin resistance in T2D patients [13]. Interestingly, the increased abundance of amino acids was linked to disturbances of tRNA charging and predicted as a top canonical pathway differentiating the LBW men w/NAFLD from the two other groups. Finally, when integrating metabolites and lipids in a global correlation network, two distinct communities were identified, suggesting interactions between key metabolite and lipid patterns potentially involved in NAFLD pathogenesis in the LBW subjects.

Four metabolites were differentially abundant in the LBW (all) subjects as compared with the NBW group, including higher levels of leucine, alpha-tocopherol, and cholesterol, together with lower levels of hippuric acid. Interestingly, increased levels of both leucine and cholesterol have been associated with low birth weight as well as with a higher risk of T2D [14,28,29]. Furthermore, elevated concentrations of total plasma amino acids have previously been associated with both NAFLD and insulin resistance [10]. The LBW subjects w/NAFLD had increased abundance of serum amino acids compared with the LBW subjects w/o NAFLD and the NBW controls, possibly reflecting impaired protein translation and/or degradation in the liver, and to a lesser extent increased muscle protein catabolism. In response to a high-fat overfeeding challenge, we have similarly shown elevated plasma levels of total amino acids, including citrulline, in young LBW men [30].

Both ornithine and citrulline are critical intermediates of the urea cycle, which primarily takes place in the mitochondrial matrix of hepatocytes. It has been documented, that NAFLD affects plasma levels of urea-cycle intermediates, and that NAFLD with mild fibrosis has been associated with high citrulline and low ornithine concentrations [27]. In contrast, low citrulline and high ornithine plasma levels are seen in severe fibrosis, which suggests differentiating signatures between stages of fatty liver disease [27]. Studies have furthermore shown evidence of NAFLD being associated with decreased urea-cycle enzyme levels and activities resulting in hyperammonemia and impaired urea synthesis [31]. Our finding of increased abundance of both ornithine and citrulline could suggest, on the one hand, increased urea-cycle activity in the LBW subjects w/NAFLD, representing a compensatory mechanism to remove toxic ammonia and preserve hepatic function during the early stages of liver injury. On the other hand, elevated levels of ornithine and citrulline might arise from an impaired degradation of amino acids, suggesting inhibited urea production and liver function. Finally, increased citrulline can also result from an increased NO cycle activity, which can be protective against or promote NAFLD depending on the nitric oxide synthase (NOS) isoforms from which it is generated [32].

Elevated concentrations of plasma tyrosine have previously been associated with lower birth weight [14]. In the current study, tyrosine was among the metabolites with the greatest significant differential (increased) abundance seen between the LBW subjects w/NAFLD and both the LBW w/o NAFLD and the NBW individuals. This finding is confirmed by other studies showing plasma tyrosine levels being positively associated with hepatic steatosis [33,34], as well as with insulin resistance in individuals with NAFLD [35], and is also in line with our finding of increased insulin resistance (HOMA-IR) in the LBW subjects w/NAFLD. Using a machine-learning approach, a study found tyrosine to be included in a panel of eight plasma metabolites which could distinguish individuals with NAFLD, NASH, as well as control subjects from each other, exhibiting increased tyrosine levels at the two disease states compared with controls [36]. A large prospective study reported elevated tyrosine levels to be associated with reduced insulin sensitivity and a corresponding increased risk of T2D [37]. Interestingly, a Mendelian randomization analysis showed that NAFLD, predicted from seven known NAFLD-susceptibility genetic variants, was strongly associated with elevated plasma tyrosine levels [38]. In the current study, the presence of NAFLD in the LBW subjects was not associated with any increased NAFLD genetic susceptibility as determined from five major NAFLD genetic variants, suggesting that NAFLD in the LBW men may be due to non-genetic factors, including an adverse intrauterine environment [3].

A pathway analysis was performed to understand biological processes associated with differentially abundant metabolites between the LBW subjects w/NAFLD compared with the other groups. In this analysis, the tRNA charging/aminoacyl-tRNA pathway was identified as the prime downregulated canonical pathway among the LBW subjects w/NAFLD. tRNA charging is essential for protein synthesis, and disturbance/dysfunction in this process is associated with T2D and aging [39,40]. Previously, we have found reduced expression levels of selected key insulin signaling proteins in adipose tissue from young LBW men, suggesting impaired protein translation and possibly contributing to development of insulin resistance [41,42]. tRNA charging is among pathways with significant differences between individuals with early-stage NAFLD and healthy controls [43]. Furthermore, studies have suggested tRNA fragments as promising biomarkers for liver fibrosis in NAFLD, and tRNA charging was also identified to be among the most differentially regulated pathways for prediction of NAFLD severity in a multi-omics study [44,45]. The pathway analysis also indicated that the mitochondrial UCP2 pathway, as well as the nutrient sensing mTOR signaling pathway, were inhibited in the LBW w/NAFLD subjects compared with the NBW controls. The UCP2 pathway is involved in the control of uncoupling of oxidative phosphorylation and ATP generation, as well as in protection against mitochondrial oxidative stress and reactive oxygen species (ROS) generation. Reduced protection against ROS may promote inflammation and fibrosis representing suspected key pathogenic mechanisms in the development of NAFLD. Conversely, the impaired mTOR signaling pathway in the LBW subjects w/NAFLD is more likely to represent a compensatory mechanism. Downregulation of the mTORC1 protein complex has been suggested to protect mice from developing NAFLD and NASH [46].

A total of 56 significant lipids were found across the four differential abundance analyses. Of these, TGs and PCs dominated the lipid species showing increased levels in the LBW subjects w/NAFLD compared with the LBW w/o NAFLD and NBW groups. The cholesteryl esters (CE) lipid subclass showed the largest decrease in abundance in the LBW w/NAFLD subjects compared with the groups w/o NAFLD, with only cholesteryl linoleate (CE(18:2)) being significantly lower after FDR correction when comparing the LBW w/NAFLD subjects with the LBW w/o NAFLD group. Investigations of the liver lipidome have revealed that TGs and CEs are among the lipid subclasses showing the most differential levels across disease states from simple steatosis to cirrhosis [47]. This included increased levels of TG species in NASH and steatosis compared with normal liver samples, and lower CE(18:2) in NASH compared with simple steatosis [47]. Importantly, a similar finding has been shown in serum samples, where a decrease in CEs and an increase in total TGs were the most pronounced alterations in NAFLD compared with control individuals [48]. We previously documented increased lipolysis in young, lean LBW individuals, suggesting increased flow of free fatty acids to the liver providing substrate for TG synthesis [49]. The increased abundance of serum TG species in the LBW w/NAFLD subjects may suggest hepatic overproduction and secretion of TG in the form of very low density lipoproteins (VLDL), possibly as a compensatory mechanism to avoid further hepatic lipid accumulation. Increased circulating phospholipids (PCs) in serum, and specifically lysophosphatidylethanolamine (LPE), have also been suggested as a potential biomarker to predict the risk of progressing from NAFLD to NASH [50].

Our finding of TGs and PCs with relatively fewer double bonds, reflecting increased saturation, as well as shorter carbon-chain length, in the LBW subjects w/NAFLD compared with LBW and NBW subjects without NAFLD, is in agreement with previous studies of NAFLD individuals [12,13,48,51]. Notably, TGs with a shorter carbon-chain length and lower double-bond content were previously reported to be associated with an increased risk of later development of T2D and metabolic syndrome [13,23]. Interestingly, a recent paper found this structural pattern of TGs to be present in the transition from NAFLD to NASH, showing increased TGs with short carbon-chain length and few double bonds in NASH subjects [7]. Thus, our data suggest that not only the increased abundance of TGs and PCs in the LBW subjects w/NAFLD but also their structure are of importance for the pathogenesis of liver disease and associated with an increased risk of T2D development.

Finally, a correlation-based network analysis was performed in which a NBW reference network with combined metabolite–lipid patterns was created. Mapped onto the network were the key differentially abundant lipids and metabolites, identified from comparing both the LBW individuals w/ and w/o NAFLD with the NBW controls. Two distinct communities were identified. Community 1 included triglycerides with higher molecular weight and polyunsaturation, in addition to alpha-tocopherol and cholesterol, representing two of the most prominent component loadings of PC2, which separated the LBW w/o NAFLD group from the NBW controls. Community 2 consisted of the PC1 component loadings ornithine, tyrosine, and citrulline, which separated the LBW w/NAFLD group from the two other groups and, furthermore, correlated with hepatic fat content and NAFLD status. In particular, triglycerides with shorter carbon-chain length and fewer double bonds were observed in community 2, corresponding to the pattern seen for the LBW subjects w/NAFLD in the structural analysis. Interestingly, 67% of the identified differentially abundant metabolites and lipids belonged to one of the two central communities. The results collectively point towards a tight co-regulation among differentially abundant metabolites and lipids in the LBW w/NAFLD group, suggesting that these metabolites are likely to share a common pathophysiological pathway.

This study has its strength in the deep-phenotyping and careful standardization of the study participants. The use of untargeted metabolomics and lipidomics is truly hypothesis generating and allows for discovery of novel circulating markers associated with NAFLD pathogenesis among LBW individuals. As the data are cross-sectional, interpretations of this study are only applicable to the specific timepoint. Our post hoc evaluation of the subgroup of LBW individuals w/NAFLD included only five individuals. None of the NBW individuals had screen-detected NAFLD, and thus we were not able to evaluate the independent effect of birth weight in the presence of NAFLD. Therefore, we suggest further validation in future larger studies including a group of NBW individuals with NAFLD.

## 5. Conclusions

The observed differences in metabolomics and lipidomics between the LBW and the NBW subjects in this study appeared mainly to be driven by the LBW subjects with overt NAFLD exhibiting elevated serum levels of amino acids, as well as increased abundance of more-saturated, shorter-chained serum phosphatidylcholines and triglycerides. These differences collectively reflect impaired tRNA charging, altered urea cycling and insulin resistance, and support the hypothesis that elevated hepatic fat content and NAFLD contribute to the increased risk of developing T2D in LBW subjects.

## Figures and Tables

**Table 1:** Clinical characteristics of the NBW, LBW w/o NAFLD, and LBW w/NAFLD groups.

	NBW	LBW		ANOVA
	(*n* = 22)	w/o NAFLD (*n* = 21)	w/NAFLD (*n* = 5)	*p*-Value
Birth weight (g)	3804 (±172)	2787 (±176) ^£^	2800 (±187) ^#^	**<0.001**
Age (years)	37.6 (±1.1)	37.8 (±0.9)	37.5 (±1.3)	0.83
Height (cm)	184.0 (±6.3)	178.2 (±5.7) ^£^	181.0 (±5.0)	**0.009**
Weight (kg)	84.64 (±10.63)	76.15 (±7.32) ^£^	91.22 (±6.58) *	**0.001**
BMI (kg/m^2^)	25.0 (±2.8)	24.0 (±2.2)	27.9 (±2.4) *	**0.01**
Total lean mass (DXA) (kg)	60.64 (±6.52)	55.16 (±5.18) ^£^	58.46 (±3.71)	**0.01**
Total fat mass (DXA) (kg)	20.64 (±7.97)	18.23 (±3.99)	29.48 (±6.29) ^#^ *	**0.004**
Total fat mass (DXA) (%)	24.83 (±7.46)	24.73 (±4.03)	33.32 (±5.48) ^#^ *	**0.02**
Hepatic fat (MRS) (%) ^a^	0.78 (0.58–0.90)	0.80 (0.51–1.34)	9.45 (7.44–9.54) ^#^ *	**<0.001**
F-glucose (mmol/L) ^a^	5.1 (4.9–5.2)	5.1 (4.8–5.2)	5.2 (5.1–5.8)	0.17
F-insulin (pmol/L) ^a^	53.3 (33.9–87.2)	40.7 (31.6–61.0)	101.0 (96.0–132.3) *	**0.008**
F-C-peptide (pmol/L)	702.3 (±264.7)	604.5 (±156.7)	943.6 (±353.0) *	**0.019**
F-triglyceride (mmol/L) ^a^	0.92 (0.60–1.27)	0.91 (0.73–1.12)	2.25 (1.29–5.36) ^#^ *	**0.03**
F-total cholesterol (mmol/L)	4.27 (±0.76)	4.67 (±5.39)	5.28 (±1.61)	0.05
HOMA-IR ^a^	1.62 (1.07–3.08)	1.38 (0.98–1.96)	3.30 (2.95–4.43) *	**0.006**
HGP (µmol/kg FFM/min) ^b^	6.8 (5.4–8.7)	5.7 (4.4–7.6)	6.2 (6.0–8.6)	0.53
Hepatic IR Index (insulin*HGP) ^a^	356 (214–669)	254 (166–438)	610 (591–742) *	**0.02**

Data are shown as mean ±SD for continuous variables that are normally distributed, and median (Q1–Q3) for continuous variables that do not follow a normal distribution. F = fasting; HGP = hepatic glucose production; FFM = fat free mass; IR = insulin resistance; DXA = dual-energy X-ray absorptiometry. *p*-values < 0.05 are marked in bold. ^a^ log-transformed; ^b^ differences between groups where data were not normally distributed after log transformations were calculated using the Kruskal–Wallis test; ^£^ *p* < 0.05: NBW vs. LBW w/o NAFLD; ^#^ *p* < 0.05: NBW vs. LBW w/NAFLD; * *p* < 0.05: LBW w/o NAFLD vs. LBW w/NAFLD.

**Table 2:** Differentially abundant serum metabolites (*n* = 11) denoted as log fold change (logFC) identified by four separate abundance analyses between groups (*p* ≤ 0.05). P_FDR_ ≤ 0.1 are marked in bold.

Group 1	Group 2	Metabolite	logFC	*p*-Value
LBW	NBW	Leucine	0.68	0.02
		Alpha-tocopherol	0.63	0.03
		Hippuric acid	−0.61	0.04
		Cholesterol	0.59	0.04
LBW w/NAFLD	NBW	Ornithine	1.59	**0.001**
		Tyrosine	1.33	0.007
		Citrulline	1.19	0.02
		Leucine	1.15	0.02
		Linoleic acid	−1.12	0.02
		2-Oxoisovaleric acid	1.09	0.03
		Arachidonic acid	−0.99	0.05
LBW w/o NAFLD	NBW	Hippuric acid	−0.75	0.01
		Alpha-tocopherol	0.64	0.04
LBW w/o NAFLD	NBW	Tyrosine	1.63	**0.001**
		Ornithine	1.47	**0.003**
		Citrulline	1.19	0.02
		Linoleic acid	−1.07	0.03
		Arachidonic acid	−1.06	0.03
		Alpha-ketoglutaric acid	0.99	0.04

## Data Availability

The datasets generated during and/or analyzed during the current study are available from the corresponding author on reasonable request.

## Supplementary Materials

The following supporting information can be downloaded at https://www.mdpi.com/article/10.3390/nu15071590/s1: Supplemental Methods; Figure S1: Differential abundances of metabolites between NBW and LBW subjects; Figure S2: Differential abundances of metabolites between NBW and LBW w/NAFLD subjects; Figure S3: Differential abundances of metabolites between NBW and LBW w/o NAFLD subjects; Figure S4: Differential abundances of metabolites between LBW w/o NAFLD and LBW w/NAFLD subjects; Figure S5: Differential abundances of 15 lipids between LBW and NBW subjects; Figure S6: Differential abundances of 10 lipids between LBW w/NAFLD and NBW subjects; Figure S7: Differential abundances of 37 lipids between LBW w/o NAFLD and NBW subjects; Figure S8: Differential abundances of 27 lipids between LBW w/NAFLD and LBW w/o NAFLD subjects; Figure S9: PCA plot based on the 56 differential lipids identified by the 4 differential expression sub-analyses; Table S1: Overview of the number of metabolites in the listed sub-classes; Table S2: Overview of the number of lipids in the listed sub-classes; Table S3: IPA canonical pathway analysis of tRNA charging; Table S4: IPA upstream analysis; Table S5: Lipids of interest identified by the 4 differential expression sub-analyses.
