# Supplementary material for: Circulating Metabolomic and Lipidomic Signatures Identify a Type 2 Diabetes Risk Profile in Low-Birth-Weight Men with Non-Alcoholic Fatty Liver Disease
Source: Nutrients. 2023 Mar 24;15(7):1590. doi: 10.3390/nu15071590 (PMC10096690; doi:10.3390/nu15071590)
Supplement: Supplementary file 1 [file nutrients-15-01590-s001.zip › nutrients-2243941-supplementary.pdf]

Supplemental Methods

Untargeted serum metabolomics

For the analysis, a Leco Pegasus 4D GC × GC-TOF-MS instrument (Leco Corp., St. Joseph, MI, USA) was used. The method has previously been described in detail [1]. The GC × GC-TOF-MS data were processed (i.e., alignment and normalization) using Guineu [2]. Peak areas were normalized to spiked internal standards (glutamic acid-d5, heptadecanoic acid-d33, succinic acid-d4, uric acid-2N15, and valine-d8), and median-corrected for between batch variation.

Untargeted serum lipidomics

The Folch procedure [3] was used for sample preparation with minor modifications. Briefly, serum samples were randomized and lipids were extracted from 10 µL serum using chloroform:methanol (2:1 v/v) method following addition of nine different internal standards (stable isotope labelled and non-physiological lipid species). Samples were analyzed in random order in positive and negative electrospray ionization modes using ultra-high-performance liquid chromatography quadrupole time-of-flight mass spectrometry (UHPLC-Q-TOF-MS). The UHPLC system was from Agilent Technologies (Santa Clara, CA, USA) and was used as previously described [4,5]. The lipidomics method has been cross-validated in an interlaboratory study including 31 European laboratories that used the National Institute of Standards and Technology (NIST) Standard Reference Material (SRM) 1950-Metabolites in Frozen Human Plasma [4]. The lipidomics data were pre-processed with MZmine2 [6].

The following data pre-processing steps were performed: (1) lipids were semi-quantified by normalizing the peak areas to internal standards [7], (2) systematic day-to-day variation in the measurements was removed by median batch correction [8], (3) Lipids with more than 20% missing/undetected values were omitted from subsequent analysis; The data were cross-matched with an in-house library where 252 lipids from 13 different lipid classes were identified at level 1 and 2 [5].

Omics data quality control (QC)

First, we checked for variance across each metabolite and lipid to remove the ones with zero or near zero variance (R-package nearZeroVar). Then we checked for data missingness using the R-package

DataExplorer. One value (0.01%) in the metabolomics and 11 values (0.03%) in the lipidomics data set were missing. Two negative values (0.02%) in the metabolomics and no negative values were identified in the lipidomics data set. Furthermore, we identified 3.6% ($N_{metabolite\_outliers}$=309) outliers in the metabolomics and 3.7% ($N_{lipid\_outliers}$=1368) outliers in the lipidomics data set. After the above QC, the omics data were imputed using the k-nearest neighbor algorithm (R-package knn.impute). Following this, we examined the distribution for each measurement using the normality Kolmogorov-Smirnov Test. Non-normal data were normalized (log2 transformation) followed by auto-scaling. After QC two data sets of 65 metabolites and 279 lipids were ready for further analysis.

References

[1] Pedersen HK, Gudmundsdottir V, Nielsen HB, Hyotylainen T, Nielsen T, Jensen BAH, et al. Human gut microbes impact host serum metabolome and insulin sensitivity. Nature 2016;535:376–81. https://doi.org/10.1038/nature18646.

[2] Castillo S, Mattila I, Miettinen J, Orešič M, Hyötyläinen T. Data Analysis Tool for Comprehensive Two-Dimensional Gas Chromatography/Time-of-Flight Mass Spectrometry. Anal Chem 2011;83:3058–67. https://doi.org/10.1021/ac103308x.

[3] Folch J, Lees M, Stanley GHS. A simple method for the isolation and purification of total lipides from animal tissues. J Biol Chem 1957;226:497–509. https://doi.org/10.1016/S0021-9258(18)64849-5.

[4] Bowden JA, Heckert A, Ulmer CZ, Jones CM, Koelmel JP, Abdullah L, et al. Harmonizing lipidomics: NIST interlaboratory comparison exercise for lipidomics using SRM 1950–Metabolites in Frozen Human Plasma. J Lipid Res 2017;58:2275–88. https://doi.org/10.1194/jlr.M079012.

[5] Tofte N, Suvitaival T, Ahonen L, Winther SA, Theilade S, Frimodt-Møller M, et al. Lipidomic analysis reveals sphingomyelin and phosphatidylcholine species associated with renal impairment and all-cause mortality in type 1 diabetes. Sci Rep 2019;9:16398. https://doi.org/10.1038/s41598-019-52916-w.

[6] Pluskal T, Castillo S, Villar-Briones A, Orešič M. MZmine 2: Modular framework for processing, visualizing, and analyzing mass spectrometry-based molecular profile data. BMC Bioinformatics 2010;11:395. https://doi.org/10.1186/1471-2105-11-395.

[7] Khoury S, Canlet C, Lacroix M, Berdeaux O, Jouhet J, Bertrand-Michel J. Quantification of Lipids: Model, Reality, and Compromise. Biomolecules 2018;8:174. https://doi.org/10.3390/biom8040174.

[8] Lazar C, Meganck S, Taminau J, Steenhoff D, Coletta A, Molter C, et al. Batch effect removal methods for microarray gene expression data integration: a survey. Brief Bioinform 2013;14:469–90. https://doi.org/10.1093/bib/bbs037.

Table S1. Overview of the number of metabolites in the listed sub-classes. The sub-classes are retrieved from Human Metabolome Database (HMDB).

Sub-class (HMDB)	Number of metabolites
Alcohols and polyols	1
Amines	1
Arylsulfates	1
Fatty alcohols	1
Gamma-keto acids and derivatives	1
Glycerolipids	1
Keto acids and derivatives	1
Quinone and hydroquinone lipids	1
Short-chain hydroxy acids and derivatives	1
Steroids and steroid derivatives	1
Tricarboxylic acids and derivatives	1
Unknown	1
Alpha hydroxy acids and derivatives	2
Phenylpropanoic acids	2
Benzene and substituted derivatives	3
Dicarboxylic acids and derivatives	3
Indolyl carboxylic acids and derivatives	3
Beta hydroxy acids and derivatives	4
Carbohydrates and carbohydrate conjugates	4
Fatty acids and conjugates	15
Amino acids, peptides, and analogues	17
Total	**65**

Table S2. Overview of the number of lipids in the listed sub-classes. The sub-classes are retrieved from Human Metabolome Database (HMDB).

Sub-class (HMDB)	Abbreviation	Number of Metabolites
Diacylglycerol	DG	2
Phosphatidic acid	PA	3
Cholesteryl ester	CE	4
Fatty acid	FA	5
Lysophosphatidylethanolamine	LPE	8
Phosphatidylglycerol	PG	10
Ceramide	CER	18
Sphingolipids	SM	28
Lysophosphatidylcholines	LPC	30
Phosphatidylethanolamine	PE	31
Phosphatidylcholines	PC	67
Triglyceride	TG	73
Total		**279**

Table S3. IPA canonical pathway analysis of tRNA charging. The table shows output from the 4 different sub-analyses (shown in the header). Red indicates upregulated values and green indicates downregulated values compared to IPA database values.

**Metabolite**	**LBW vs. NBW**		**LBW w/o NAFLD vs. NBW**		**LBW w/ NAFLD vs. NBW**		**LBW w/ NAFLD vs. LBW w/o NAFLD**	
	**Expr Log Ratio**	**Expr FDR (q-value)**	**Expr Log Ratio**	Expr FDR (q-value)	Expr Log Ratio	Expr FDR (q-value)	Expr Log Ratio	Expr FDR (q-value)
Glycine	−0.211	9.19E-01	−0.083	9.66E-01	−0.750	5.90E-01	−0.667	5.80E-01
Alanine	−0.401	8.31E-01	−0.529	7.57E-01	0.134	9.29E-01	0.663	5.80E-01
Glutamic acid	−0.006	9.84E-01	−0.114	9.66E-01	0.446	7.61E-01	0.559	5.80E-01
Isoleucine	0.222	9.19E-01	0.113	9.66E-01	0.678	6.47E-01	0.565	5.80E-01
Leucine	0.680	7.04E-01	0.569	7.57E-01	1.146	2.97E-01	0.577	5.80E-01
Methionine	−0.252	9.05E-01	−0.343	8.69E-01	0.129	9.29E-01	0.472	6.51E-01
Phenylalanine	−0.223	9.19E-01	−0.393	8.69E-01	0.493	7.12E-01	0.886	4.81E-01
Proline	0.119	9.65E-01	0.026	1.00E00	0.509	7.12E-01	0.483	6.50E-01
Threonine	0.051	9.72E-01	0.118	9.66E-01	−0.228	9.19E-01	−0.346	7.88E-01
Tryptophan	0.118	9.65E-01	−0.019	1.00E00	0.695	6.44E-01	0.714	5.70E-01
Tyrosine	0.016	9.72E-01	−0.297	8.69E-01	1.329	2.29E-01	1.626	6.73E-02
Valine	0.290	9.05E-01	0.291	8.69E-01	0.286	8.88E-01	−0.006	9.93E-01

Table S4. IPA upstream analysis. The table shows output from the 2 out of the 4 different sub-analyses (shown in the header). A negative z-score indicates inhibition, and a positive z-score indicates activation.

**Upstream regulator**	**LBW w/o NAFLD vs. NBW**		**LBW w/ NAFLD vs. NBW**	
	**Activation z-score**	p-value of overlap	Activation z-score	p-value of overlap
mTOR			−2	2.35E-05
UCP2	−1	3.03E-04	−1	3.03E-04

Table S5. Lipids of interest identified by the 4 differential expression sub-analyses, with a total of 56 lipids being differential (P≤0.05 and *P_{FDR}≤0.1 threshold) (next page).

Group1	Group2	Lipids	logFC	P-value	Adj P-val (FDR)
LBW	NBW	GlcCer(42:1)	0.6673	0.0223	0.7073
		PC(34:1)	0.5754	0.0488	0.7073
		PC(34:3)	-0.599	0.0403	0.7073
		PC(42:6)	0.8522	0.0035	0.6453
		PE(37:5)	-0.5774	0.048	0.7073
		PE(39:4)	0.6429	0.0277	0.7073
		PE(41:7)	0.6575	0.0244	0.7073
		SM(34:1)	0.6149	0.0352	0.7073
		SM(36:2)	0.8271	0.0046	0.6453
		TG(44:3)	-0.7107	0.015	0.7073
		TG(46:3)	-0.5887	0.0438	0.7073
		TG(52:7)	-0.6415	0.0281	0.7073
		TG(53:6)	-0.6661	0.0226	0.7073
		TG(58:12)	-0.6163	0.0348	0.7073
		TG(60:13)	-0.5966	0.0411	0.7073
LBW w/ NAFLD	NBW	CE(18:2)	-16.194	0.0011	0.3108
		CE(20:4)	-13.491	0.0066	0.5963
		PA(43:0)	-11.647	0.019	0.8848
		PC(32:1)	10.418	0.036	0.9761
		PC(34:1)	13.061	0.0085	0.5963
		PC(42:6)	13.157	0.0081	0.5963
		PE(37:4)	12.541	0.0116	0.646
		PG(38:1)	-0.9747	0.0497	0.9761
		SM(36:2)	0.9901	0.0462	0.9761
		TG(52:2)	10.294	0.0382	0.9761
LBW w/o NAFLD	NBW	GlcCer(42:1)	0.7078	0.0207	0.3059
		LPC(14:0)	-0.6033	0.0486	0.3661
		PC(28:0)	-0.6281	0.04	0.3434
		PC(30:1)	-0.663	0.0302	0.3065
		PC(32:2)	-0.6891	0.0243	0.3059
		PC(32:3)	-0.6544	0.0324	0.3065
		PC(33:3)	-0.6187	0.0431	0.3434
		PC(34:3)	-0.6589	0.0312	0.3065
		PC(34:4)	-0.6195	0.0428	0.3434
		PC(36:3)	-0.7288	0.0172	0.3059
		PC(36:6)	-0.6711	0.0282	0.3065
		PC(36:7)	-0.7149	0.0194	0.3059
		PC(42:6)	0.7419	0.0153	0.3059
		PE(37:5)	-0.7503	0.0142	0.3059
		PE(39:4)	0.6201	0.0426	0.3434
		PE(39:6)	-0.6349	0.0379	0.3411
		PE(41:7)	0.7266	0.0175	0.3059
		SM(34:1)	0.7257	0.0177	0.3059
		SM(36:2)	0.7883	0.01	0.3059
		SM(44:2)	-0.6858	0.0249	0.3059
		TG(40:1)	-0.7307	0.0169	0.3059
		TG(44:1)	-0.6625	0.0303	0.3065
		TG(44:2)	-0.7049	0.0212	0.3059

		TG(44:3)	-0.8996	0.0033	0.3059
		TG(46:2)	-0.6845	0.0252	0.3059
		TG(46:3)	-0.7841	0.0104	0.3059
		TG(47:2)	-0.6885	0.0244	0.3059
		TG(48:3)	-0.697	0.0227	0.3059
		TG(52:7)	-0.734	0.0164	0.3059
		TG(53:6)	-0.7417	0.0153	0.3059
		TG(54:7)	-0.6643	0.0299	0.3065
		TG(54:8)	-0.7068	0.0208	0.3059
		TG(54:9)	-0.6901	0.0241	0.3059
		TG(58:12)	-0.8453	0.0057	0.3059
		TG(59:10)	-0.6039	0.0483	0.3661
		TG(60:13)	-0.8533	0.0053	0.3059
		TG(62:11)	-0.6523	0.033	0.3065
LBW w/ NAFLD	LBW w/o NAFLD	CE(18:2)	-19.649	1e-4	0.0229*
		CE(20:4)	-1.405	0.0049	0.3833
		PA(43:0)	-13.077	0.0088	0.3833
		PA(44:1)	1.051	0.0351	0.4528
		PC(28:0)	12.416	0.0128	0.3833
		PC(30:0)	10.952	0.0281	0.4528
		PC(30:1)	0.9866	0.048	0.4528
		PC(32:1)	11.316	0.0233	0.4336
		PC(33:1)	10.123	0.0424	0.4528
		PC(34:4)	10.034	0.0443	0.4528
		PC(34:5)	13.419	0.0072	0.3833
		PC(36:3)	11.353	0.0229	0.4336
		PC(36:5)	12.399	0.0129	0.3833
		PC(36:6)	1.371	0.006	0.3833
		PC(38:8)	12.006	0.0161	0.3957
		PE(37:4)	12.679	0.011	0.3833
		PE(43:6)	10.378	0.0375	0.4528
		PG(40:4)	-10.664	0.0326	0.4528
		TG(44:3)	0.9826	0.0489	0.4528
		TG(46:3)	10.161	0.0417	0.4528
		TG(48:2)	11.356	0.0228	0.4336
		TG(54:9)	0.9829	0.0488	0.4528
		TG(57:5)	11.161	0.0253	0.4407
		TG(58:12)	11.905	0.017	0.3957
		TG(60:12)	10.829	0.03	0.4528
		TG(60:13)	13.348	0.0075	0.3833
		TG(62:11)	12.293	0.0137	0.3833

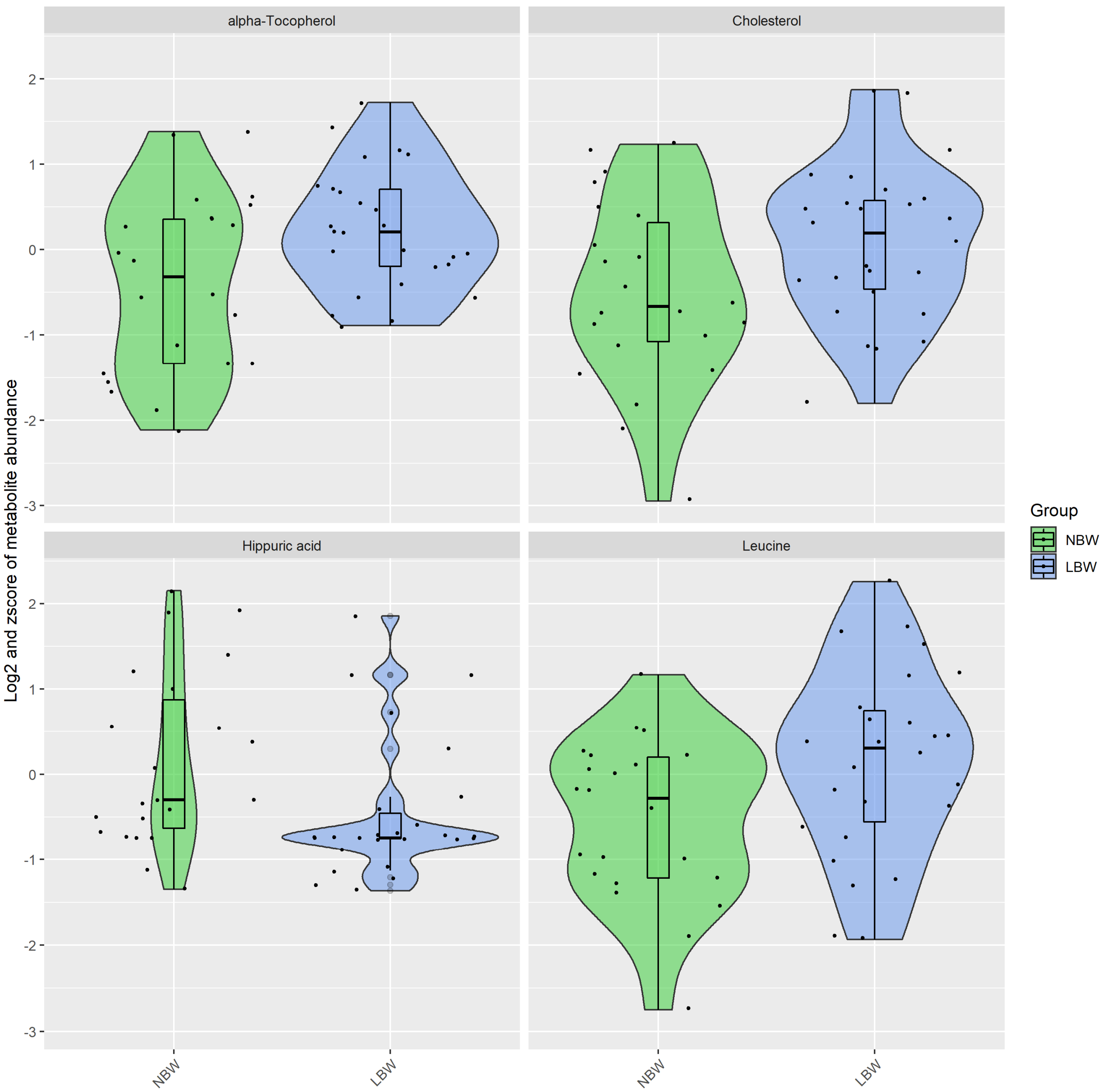

Figure S1. Differential abundances of metabolites between NBW and LBW (all) subjects. Not corrected for multiple testing (P<0.05).

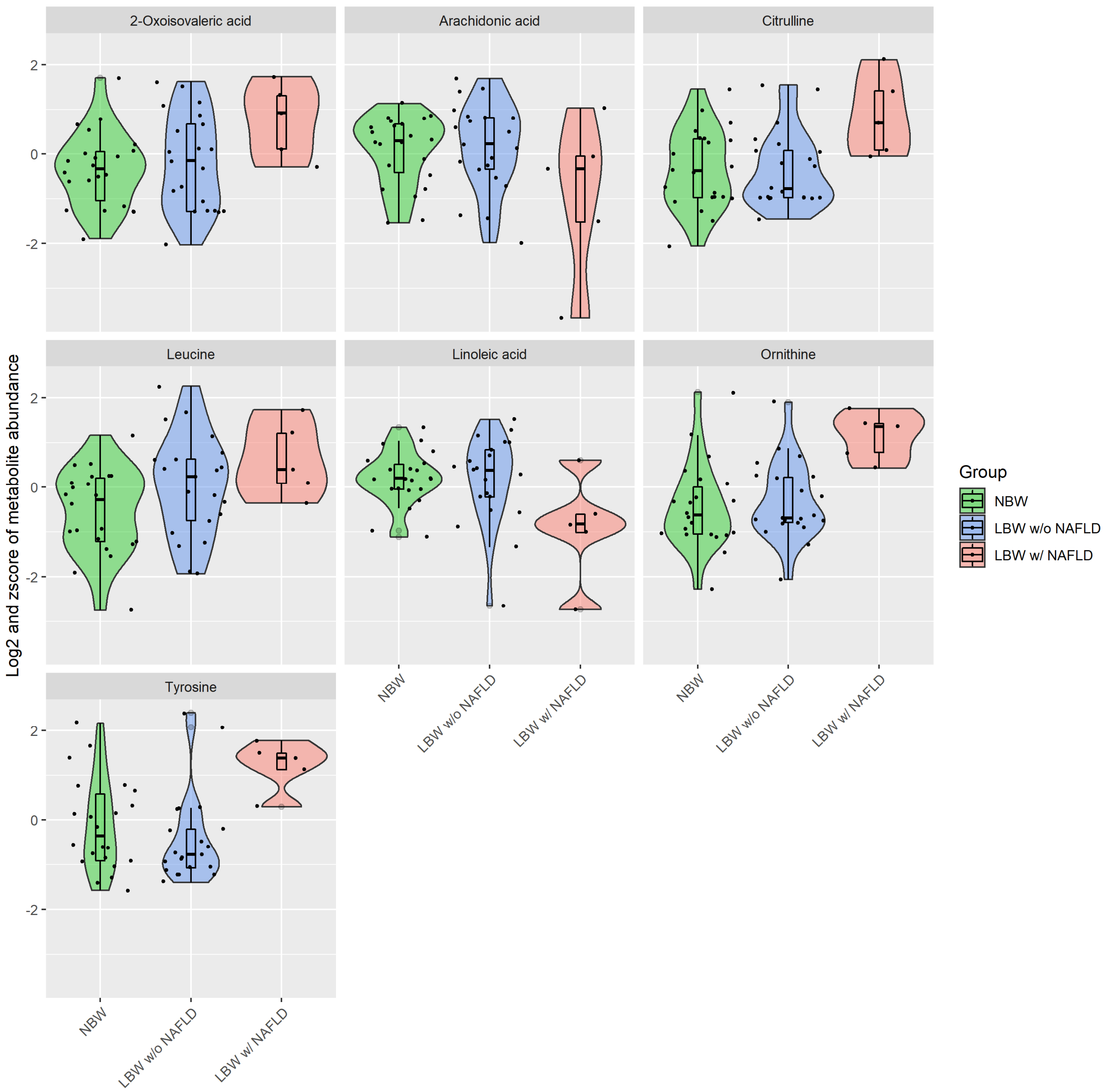

Figure S2. Differential abundances of metabolites between NBW vs. LBW w/ NAFLD subjects. A total of 6 metabolites ($P<0.05$) and ornithine ($P_{FDR}=0.08$) were differentially abundant.

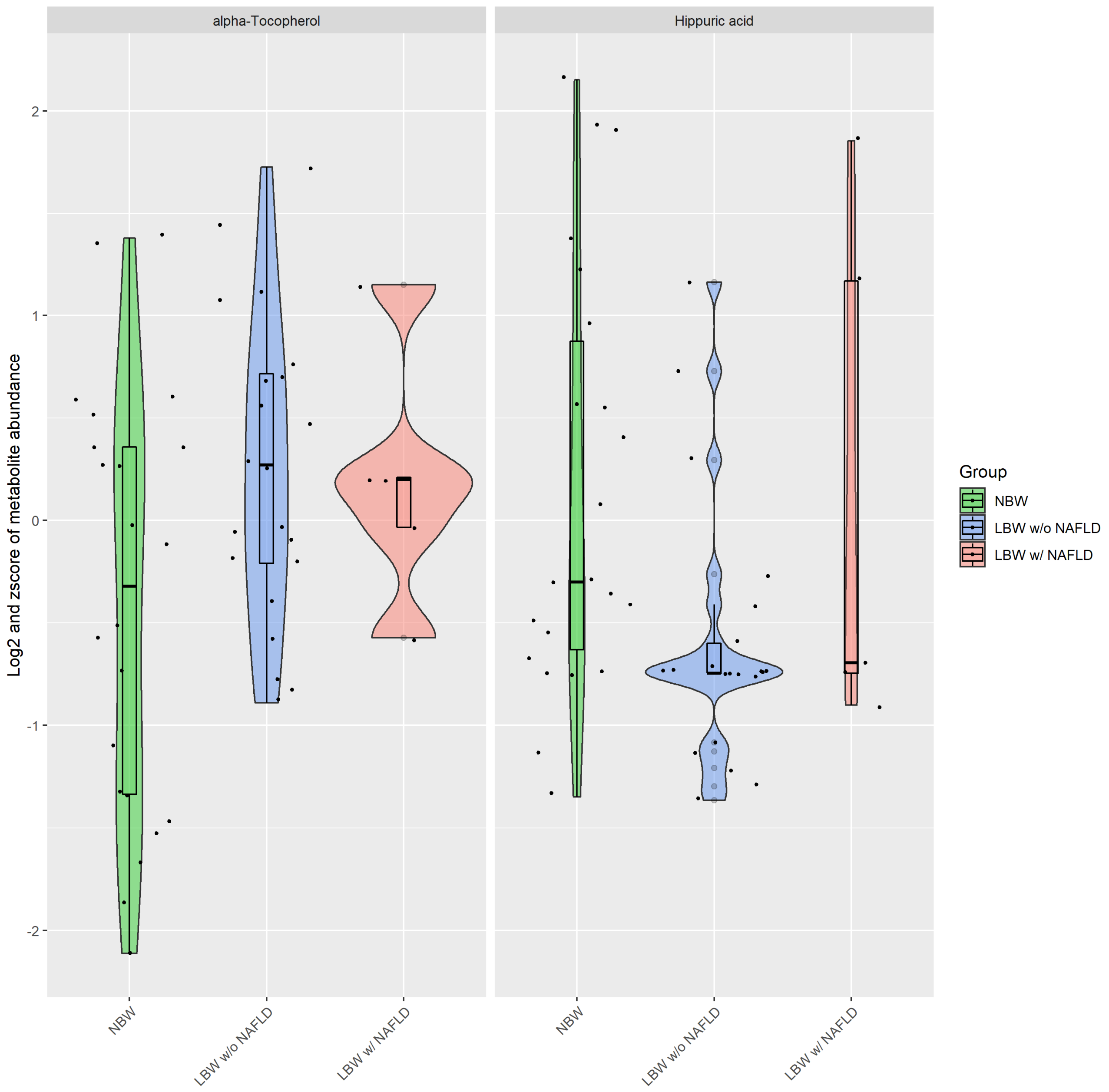

Figure S3. Differential abundances of metabolites between NBW and LBW w/o NAFLD subjects. Not corrected for multiple hypothesis testing (P<0.05).

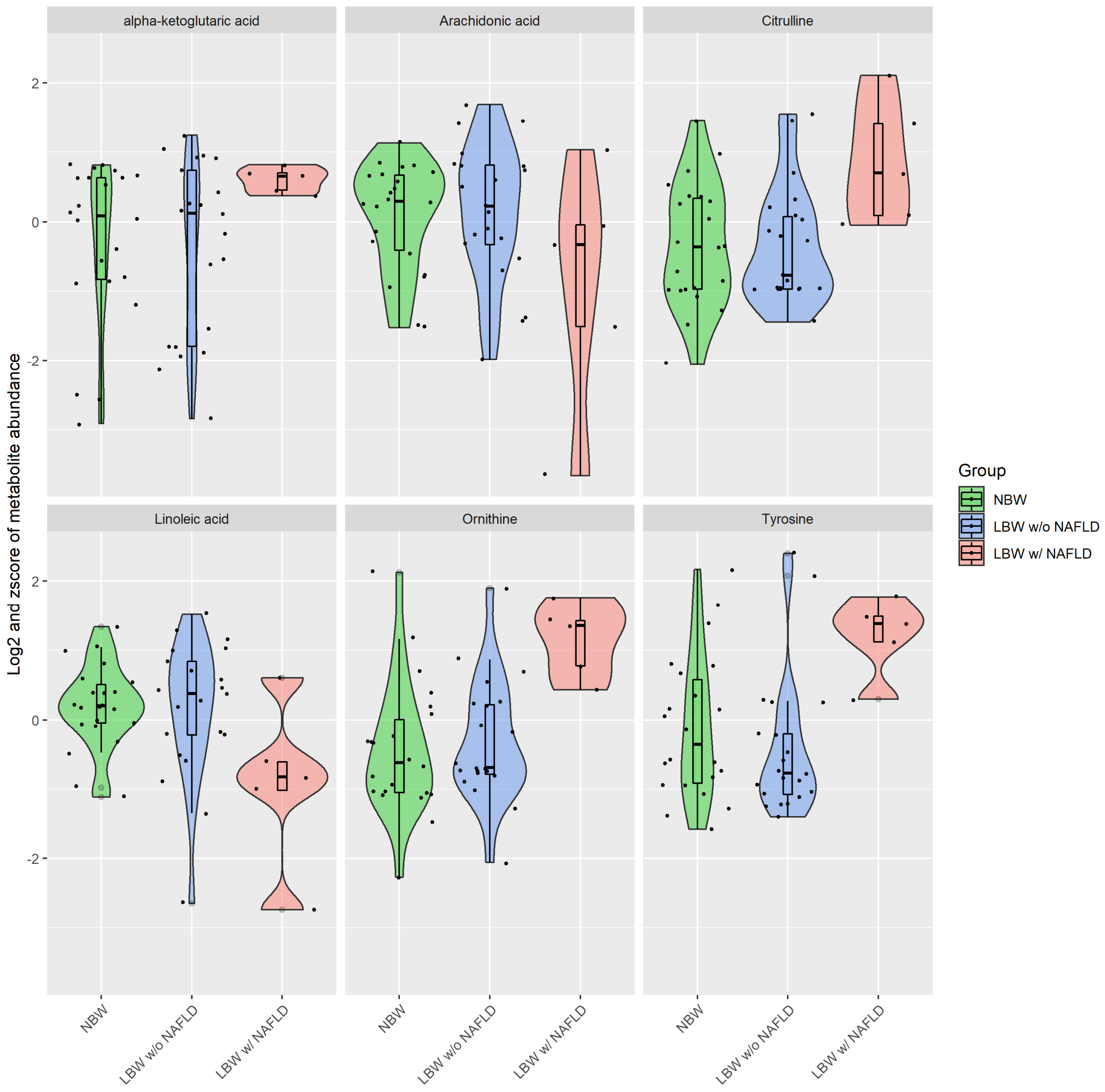

Figure S4. Differential abundances of metabolites between LBW w/o NAFLD vs. LBW w/ NAFLD subjects. A total of 5 lipids (P<0.05) and tyrosine (P_{FDR}=0.07) were differentially abundant between the two groups.

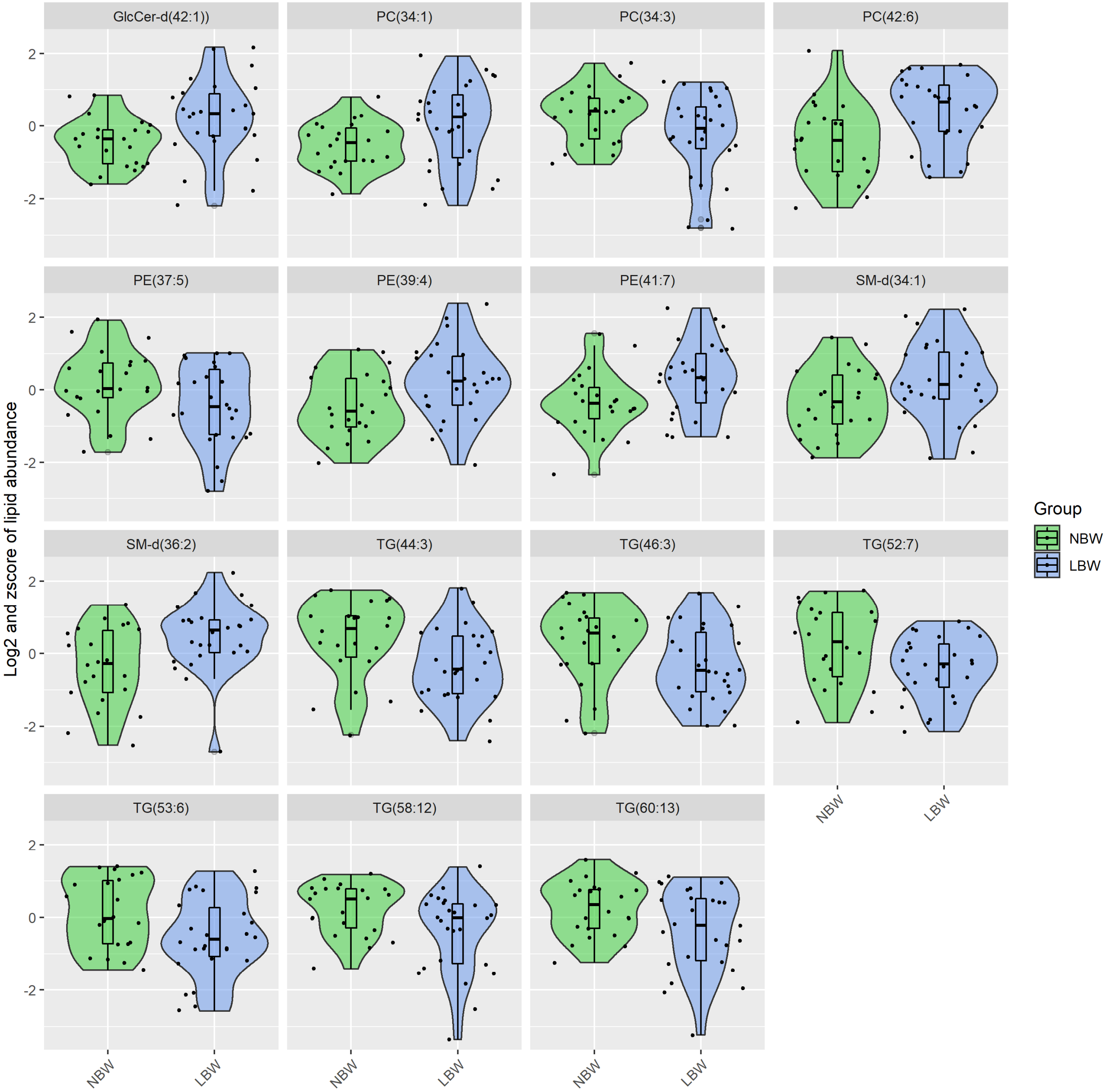

Figure S5. Differential abundances of 15 lipids between LBW (all) and NBW subjects. Not corrected for multiple testing (P<0.05).

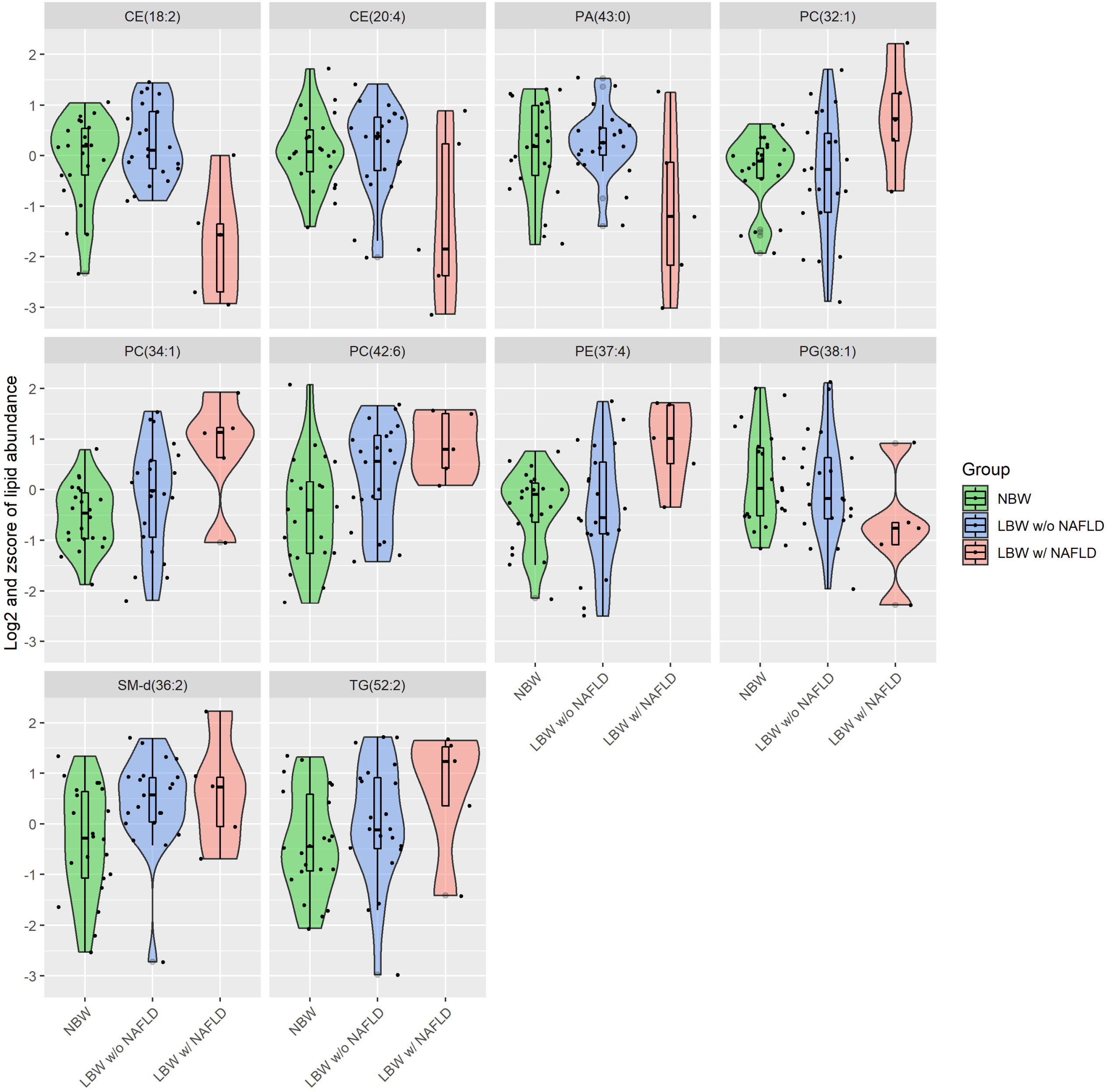

Figure S6. Differential abundances of 10 lipids between LBW w/ NAFLD vs. NBW. Not corrected for multiple testing (P<0.05).

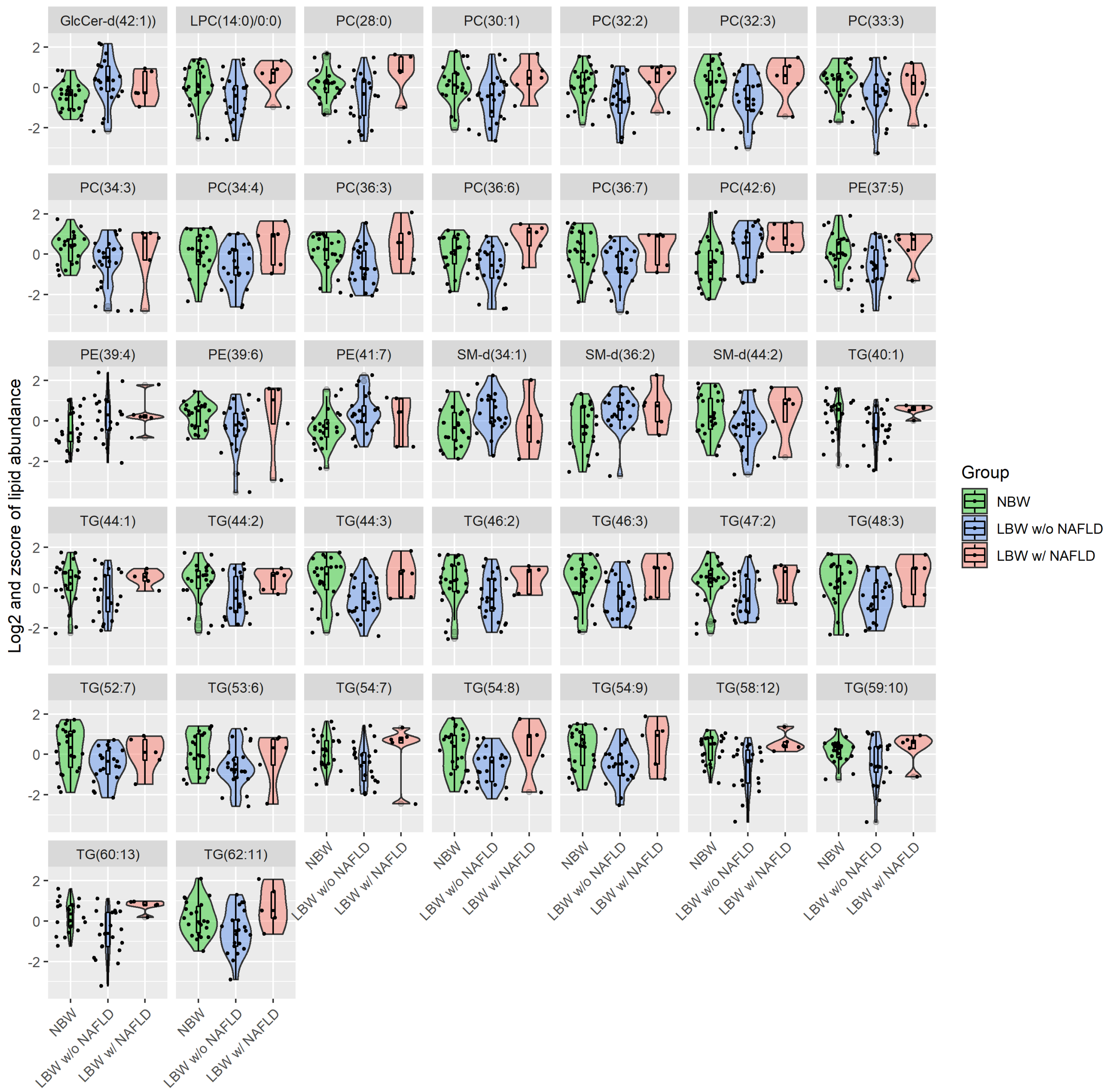

Figure S7. Differential abundances of 37 lipids between LBW w/o NAFLD vs. NBW. Not corrected for multiple testing (P<0.05).

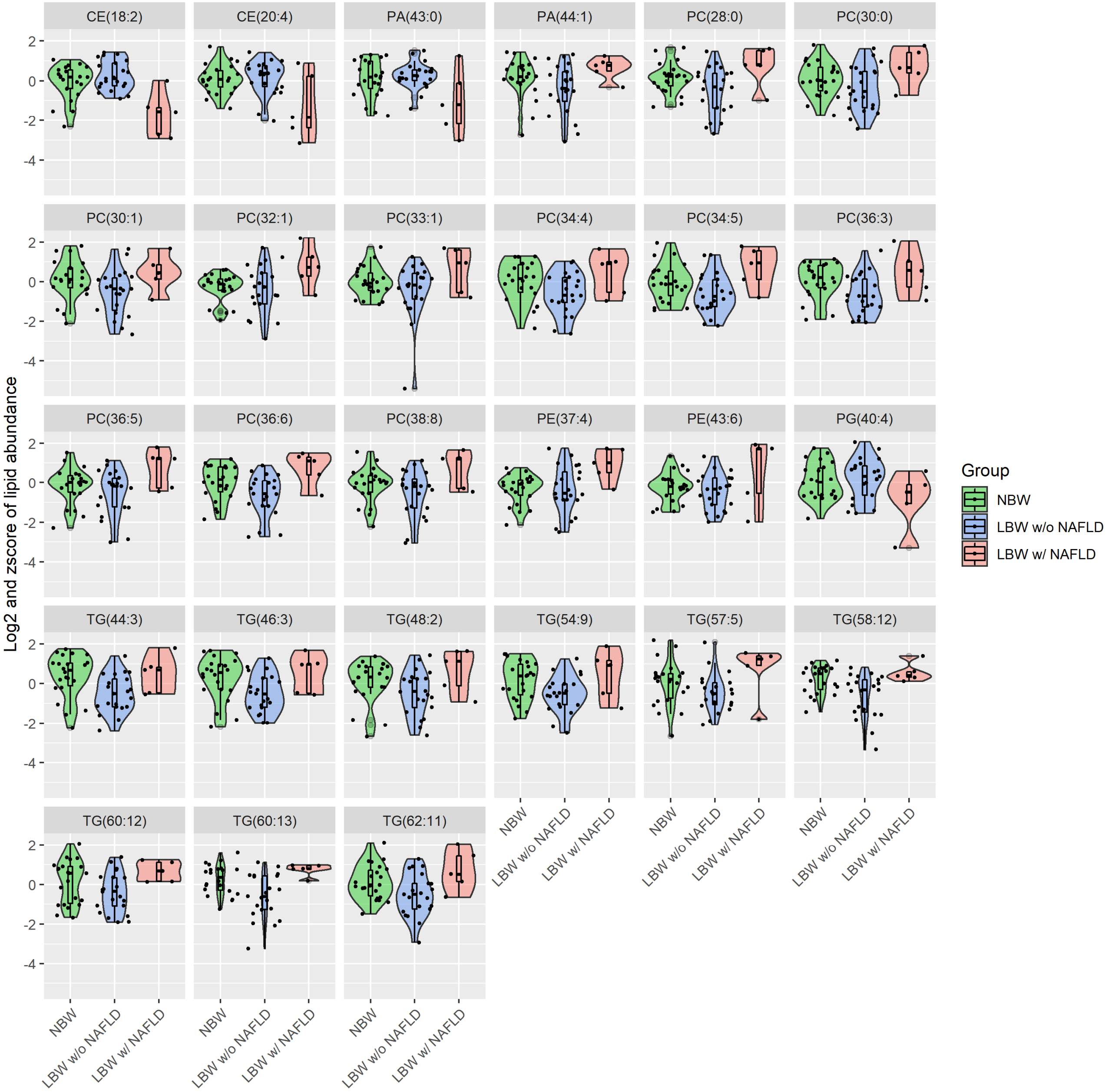

Figure S8. Differential abundances of 27 lipids between LBW w/ NAFLD vs. LBW w/o NAFLD subjects. Not corrected for multiple testing (P<0.05).

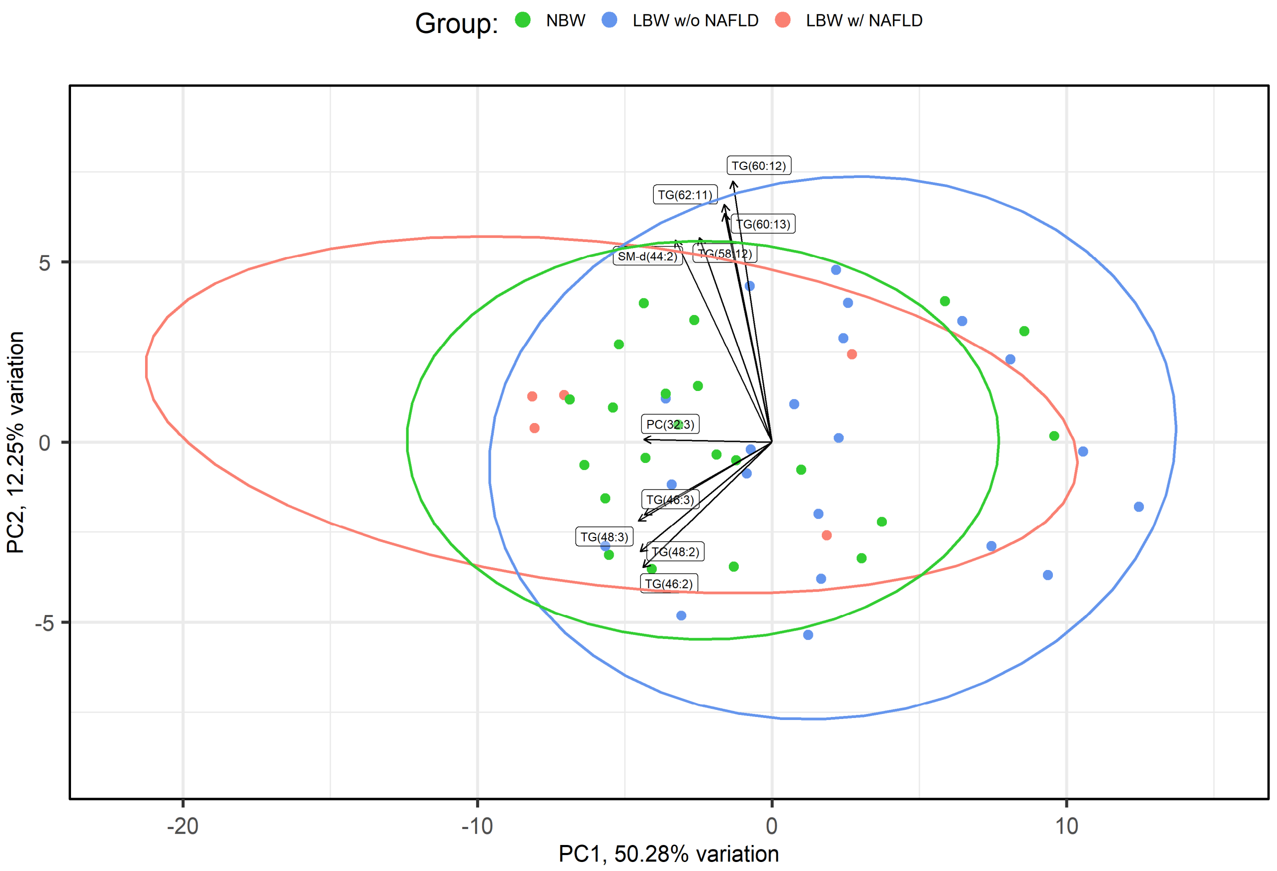

Figure S9. PCA plot based on the 56 differential lipids identified by the 4 differential expression sub-analyses.
